# Association between free fatty acids and vascular calcification in CKD patients

**DOI:** 10.3389/fphys.2026.1908095

**Published:** 2026-07-22

**Authors:** Xingtong Dong, Na Lin, Congcong Zhu, Wenjing Fu, Aihua Zhang

**Affiliations:** Department of Nephrology, Xuanwu Hospital, Capital Medical University, Beijing, China

**Keywords:** abdominal aortic calcification, cardiovascular disease, chronic kidney disease, free fatty acids, vascular calcification

## Abstract

**Purpose:**

As an independent risk factor for cardiovascular events in patients with chronic kidney disease (CKD), the mechanism of vascular calcification (VC) remains unclear, and current treatments targeting known mechanisms have still failed to reduce its occurrence. The relationship between free fatty acids (FFAs) and vascular calcification has been confirmed in general population in recent years, but not in CKD patients. Therefore, we investigated the association between free fatty acids and vascular calcification in CKD patients.

**Methods:**

A total of 103 patients diagnosed with CKD stage 3–4 were included in this cross-sectional study. The severity of VC in this population was evaluated by abdominal aortic calcification score (AACS). Univariate binary logistic regression analysis was used for univariate analysis. Parameters with *p* < 0.1 in univariate analysis and established risk factors were considered potential confounders and were entered into multivariable logistic regression analysis. The discriminatory ability of FFAs on the presence of AAC was analyzed by receiver operating characteristic curves (ROC) according to the area under the curve (AUC).

**Results:**

The serum FFAs levels were significantly increased in the group with AAC scores ≥ 5 group [0.67 (0.65, 1.13) mmol/L vs. 0.62 (0.39, 0.65) mmol/L, *P*<0.001]. FFAs levels were independently associated with AAC severity (OR=1.269, *P* = 0.012, 95% CI:1.053-1.528). FFAs levels showed the biggest AUC of 0.868, with a sensitivity of 71.4% and a specificity of 94.1% and the optimal threshold was 0.665 mmol/L.

**Conclusion:**

Elevated serum FFAs are associated with vascular calcification severity in patients with CKD stage 3–4.

## Introduction

Chronic kidney disease (CKD) ranks the ninth leading cause of death globally, accounting for 1·48 million deaths in 2023. Meanwhile, 788 million people aged 20 years and older were estimated to have CKD in the world, the global age-standardized prevalence of CKD in adults was 14·2% (Mark et al., 2025). By 2040, CKD is projected to become the fifth leading cause of death worldwide. However, cardiovascular disease remains the primary cause of mortality in CKD patients, with vascular calcification (VC) having been established as an independent risk factor for cardiovascular events in this population (Mencke et al., 2024). Patients with CKD face a fourfold higher risk of cardiovascular calcification (CVC) compared to healthy individuals (Carney, 2020). Vascular calcification is anatomically classified into three main types: intimal calcification, medial calcification (also known as Mönckeberg’s sclerosis, predominantly occurring in CKD patients) and cardiac valvular calcification. Emerging evidence indicates that vascular calcification represents an active, highly regulated cellular process resembling bone development (Hénaut et al., 2018). The primary pathogenic mechanisms involve dysregulation of calcium-phosphate metabolism imbalance between pro-calcific and anti-calcific factors, osteogenic transformation of vascular smooth muscle cells (VSMCs), cellular apoptosis, vascular matrix remodeling and inflammatory responses (Chen et al., 2024). Notably, VSMC osteogenic differentiation constitutes the central pathological event in vascular calcification. Despite these mechanistic insights, current clinically available therapies targeting these pathways have failed to reduce the incidence of vascular calcification in CKD patients. Consequently, further research into VC pathogenesis and the development of effective preventive strategies remain clinically imperative.

Serum free fatty acids (FFAs), also termed non-esterified fatty acids, serve as essential energy substrates and participate in diverse metabolic regulations (Bianchi, 2020). It has been reported that both patients with CKD and those undergoing dialysis exhibit elevated blood levels of FFAs (Wu et al., 2010; Chen et al., 2017). This may be attributed to reduced renal clearance of FFAs due to declining kidney function, leading to increased circulating FFA levels. Additionally, CKD is often accompanied by dyslipidemia, which further enhances lipolysis and FFA release (Xiang et al., 2017). Cellular and animal models of vascular calcification have shown that elevated FFAs promote vascular calcification through multiple pathways: FFAs can activate inflammatory pathways (eg, NF-κB) (Zhong et al., 2022), increasing the release of pro-inflammatory cytokines such as TNF-α and IL-6, which induce vascular smooth muscle cells (VSMCs) to undergo osteogenic differentiation, thereby facilitating calcium deposition. Furthermore, FFAs may mediate ferroptosis of VSMCs, contributing to the development of vascular calcification (Chen et al., 2023). However, there are few clinical investigations exploring associations of FFAs and vascular calcification in CKD populations. It was only reported in the general population that FFAs were associated with coronary artery calcification (Xin et al., 2021). Therefore, this study will systematically examine the relationship between serum FFAs and vascular calcification in CKD patients, addressing a critical knowledge gap in nephrology and cardiovascular research.

## Methods

### Subjects

**Figure 1:**
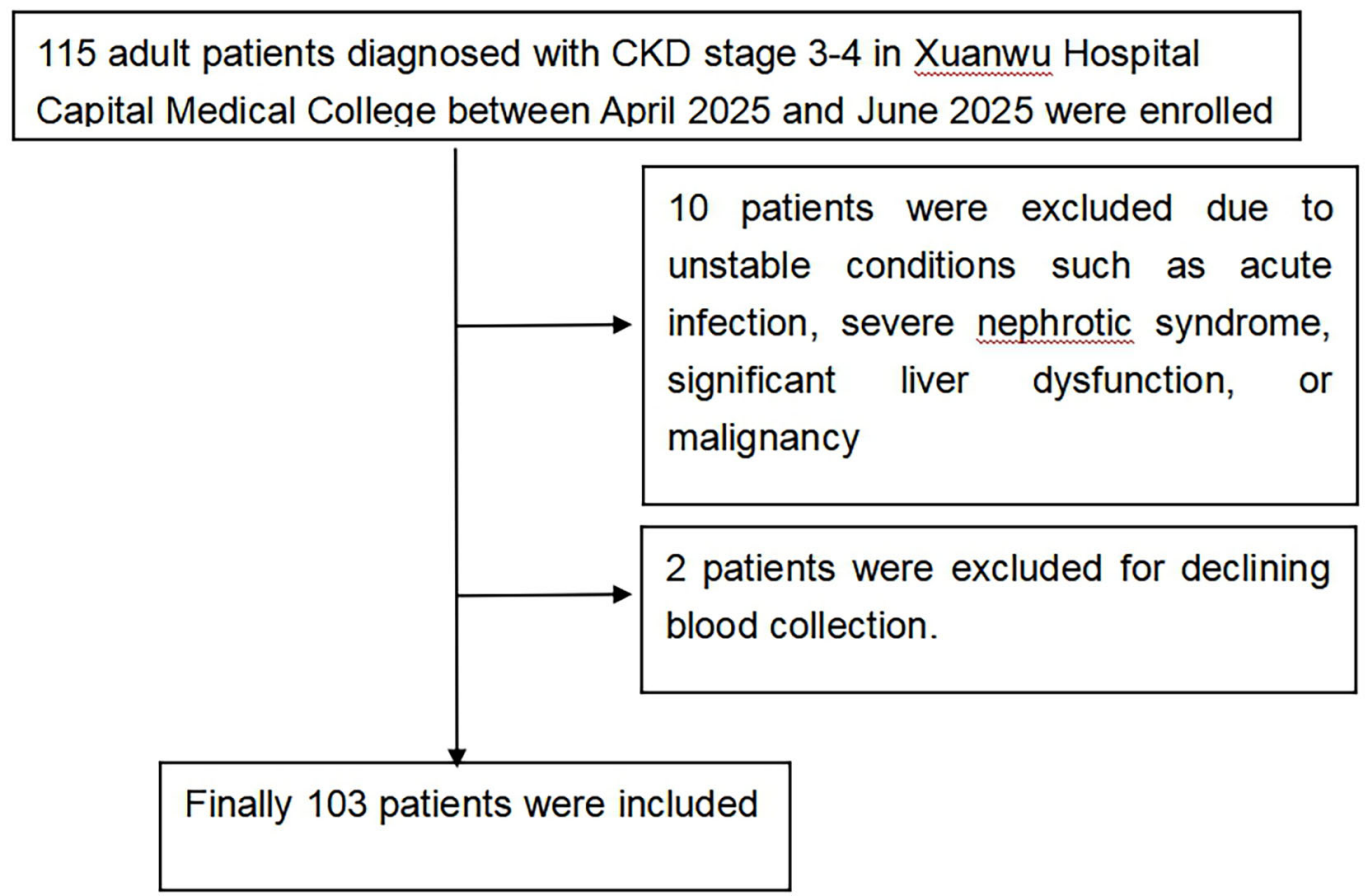
Flowchart of the study.

This cross-sectional study was conducted in Xuanwu Hospital Capital Medical University between April 2025 and June 2025. The inclusion criteria for patients were as follows: (1) over 18 years of age; (2) diagnosed with CKD stage 3-4 and (3) agreed to participate in this study. The exclusion criteria for patients were as follows: (1) malignant tumor; (2) severe nephrotic syndrome; (3) patients were in the acute phase of infection, heart failure or other comorbidities; (4) their data were incomplete. A flowchart is shown in **Figure 1**.

Finally, 103 patients in our hospital were enrolled in this study. This study was approved by the Ethics Committee of our hospital [No. (2019)131].

### Data collection

Demographic and clinical data including age, sex, causes of CKD, smoking/drinking history, medical history and the use of phosphorus binders, lipid-lowering drugs, antiplatelet drugs and vitamin D were collected. All the clinical and laboratory data such as systolic blood pressure(SBP), diastolic blood pressure(DBP), hemoglobin, estimated glomerular filtration rate (eGFR), serum creatinine, serum urea nitrogen, serum uric acid, fasting serum glucose, alanine aminotransferase (ALT), aspartate aminotransferase (AST), hemoglobin A1c (HbA1c), lipid profile including total cholesterol, triglycerides, low-density lipoprotein cholesterol (LDL-C) and high-density lipoprotein cholesterol (HDL-C), albumin, phosphate, calcium, intact parathyroid hormone (iPTH), urine total protein (UTP), C reactive protein (CRP), carbondioxide combining power (CO2CP) were collected at baseline.

### Measurement of serum FFAs

Blood samples were collected after fasting for at least 8 hours using vacuum tubes without anticoagulant. Then they were centrifuged and the serum was separated and stored at −80 °C until analysis. Serum FFAs were measured enzymatically using a Non-esterified Fatty Acid (NEFA) Assay Kit (BSBE, China) on a Roche Cobas 6000 c501 analyzer. The assay principle is based on the ACS-ACOD method, with final chromogenic detection at approximately 546 nm. All measurements were performed in duplicate according to the manufacturer’s protocol.

### Abdominal aortic calcification scoring

The abdominal aortic calcification (AAC) scoring has been widely recognized as a validated method for evaluating vascular calcification in CKD patients. In this study, patients were assessed using lateral lumbar X-ray obtained during hospitalization. Two physicians blinded to patients’ clinical information calculated the AAC scores using a 24-point scoring system developed by Kauppila et al (Kauppila et al., 1997). Calcification scores for both the anterior and posterior walls of the abdominal aorta from the first to fourth lumbar vertebrae (L1-L4) were recorded. Each vertebral segment was scored from 0 to 3: 0 = no calcification; 1 = calcification length <1/3 of the vertebral height; 2 = calcification length covering 1/3 to 2/3 of the vertebral height; 3 = calcification length >2/3 of the vertebral height. The total AAC score represented the sum of scores from L1 to L4, with a maximum possible score of 24 (≤4:no or mild calcification; 5-15:moderate calcification;16-24: severe calcification).

### Statistical analysis

Results were expressed as proportions for categorical variables. Continuous variables following a normal distribution were presented as mean ± standard deviation (SD), and comparisons between two groups were performed using the independent two-sample t-test. Continuous variables not following a normal distribution were presented as median with interquartile range (IQR), and intergroup comparisons for non-normally distributed data were conducted using the Mann-Whitney U test. Comparisons of categorical data were performed using the chi-square test. Univariate analysis was carried out using univariate binary logistic regression. Variables with a *P* value < 0.1 in the univariate regression analysis were considered potential confounding factors and were subsequently included in a multivariate logistic regression analysis. In the regression models of this study, the tolerance of each independent variable was greater than 0.1 and the variance inflation factor (VIF) was less than 10, indicating the absence of multicollinearity among the independent variables. The Hosmer-Lemeshow test was used to assess the goodness-of-fit of the model, with a *P* value > 0.05 indicating a good model fit. The discriminatory ability of FFAs for the presence of AAC was analyzed using the receiver operating characteristic (ROC) curve and the area under the curve (AUC). Statistical analyses were performed using SPSS version 26.0 and GraphPad Prism version 10.4. All tests were two-tailed, and a *P* value < 0.05 was defined as statistically significant.

## Results

### Baseline clinical characteristics

This study consisted of 103 patients with CKD stage 3-4. The primary renal diseases were composed of chronic glomerulonephritis, diabetic kidney disease (DKD), hypertensive glomerulosclerosis, other diseases such as chronic interstitial nephritis and renal diseases with unknown causes. Patients were divided into two groups based on whether their AAC score was ≥5. As **Table 1** shows, patients with moderate to severe vascular calcification were older than those with no or mild vascular calcification [68.00 (65.00, 71.00) years vs. 60.00 (49.25, 67.00) years, *P*<0.001]. In addition they also had lower levels of hemoglobin(113.89 ± 29.20 g/L vs. 131.13 ± 22.94 g/L, *P* = 0.001) and worse renal function: serum creatinine [182.00 (148.00, 279.00) μmol/L vs. 157.00 (133.25, 189.50) μmol/L, *P* = 0.045], eGFR [28.44 (17.57, 41.83) ml/min·1.73m² vs. 40.14 (29.10, 48.63) ml/min·1.73m², *P* = 0.004], serum urea [15.80 (11.92, 21.57) mmol/L vs. 11.40 (8.80, 17.26) mmol/L, *P* = 0.002] and lower levels of calcium [2.05 (2.01, 2.13)]mmol/L vs. 2.18 (2.10, 2.27) mmol/L, *P*<0.001]. Furthermore, they were more likely to have elevated SBP, fasting serum glucose, increased iPTH and a history of diabetes as well as a higher rate of antiplatelet medication. Importantly, serum FFAs levels were significantly increased in the AAC score≥5 group[0.67 (0.65, 1.13) mmol/L vs. 0.62 (0.39, 0.65) mmol/L, *P*<0.001]. Surprisingly, patients exhibited lower levels of total cholesterol [4.05 (3.24, 5.56) mmol/L vs. 4.58 (3.82, 6.19) mmol/L, *P* = 0.045] and serum LDL-C [2.30 (1.67, 3.59) mmol/L vs. 2.85 (2.19, 4.11) mmol/L, *P* = 0.019)] in this group.

**Table 1:** Baseline clinical characteristics.

Variables	Total (n=103)	AAC score(<5) (n=68)	AAC score(≥5) (n=35)	*P* value
Age (years)	65.00 (55.00, 70.00)	60.00 (49.25, 67.00)	68.00 (65.00, 71.00)	<0.001
Male/female (n)	72/31	48/20	24/11	0.833
SBP(mmHg)	131.73 ± 16.60	128.88 ± 14.84	137.26 ± 18.58	0.015
DBP(mmHg)	82.51 ± 11.22	81.34 ± 10.92	84.80 ± 11.62	0.139
CO_2_CP(vol%)	60.75 ± 8.59	61.56 ± 8.28	59.50 ± 9.05	0.309
Smoking history(yes/no)	45/58	27/41	18/17	0.256
Drinking history(yes/no)	25/78	16/52	9/26	0.806
Hemoglobin (g/L)	125.27 ± 26.40	131.13 ± 22.94	113.89 ± 29.20	0.001
eGFR (ml/min·1.73m^2^)	35.28 (22.50, 47.23)	40.14 (29.10, 48.63)	28.44 (17.57, 41.83)	0.004
Serum creatinine (μmol/L)	159.00 (138.00, 200.00)	157.00 (133.25, 189.50)	182.00 (148.00, 279.00)	0.045
Serum urea (mmol/L)	14.55 (9.43, 18.55)	11.40 (8.80, 17.26)	15.80 (11.92, 21.57)	0.002
AST (IU/L)	18.00 (14.00, 23.00)	17.50 (14.00, 23.00)	18.00 (16.00, 23.00)	0.473
ALT (IU/L)	16.00 (11.00, 24.50)	17.00 (12.00, 28.25)	13.00 (9.00, 24.00)	0.059
HbA1c (%)	5.80 (5.30, 7.15)	6.20 (5.30, 7.28)	5.80 (4.90, 6.90)	0.371
Fasting serum glucose (mmol/L)	5.34 (4.72, 6.50)	5.16 (4.62, 6.06)	5.72 (5.11, 7.59)	0.012
Serum uric acid (μmol/L)	407.00 (337.00, 458.00)	398.50 (329.50, 447.50)	423.00 (346.00, 495.00)	0.166
Serum total cholesterol (mmol/L)	4.48 (3.67, 5.78)	4.58 (3.82, 6.19)	4.05 (3.24, 5.56)	0.045
Serum triglycerides (mmol/L)	1.64 (1.14, 2.20)	1.65 (1.20, 2.25)	1.59 (1.04, 2.19)	0.662
Serum LDL-C (mmol/L)	2.66 (2.00, 3.74)	2.85 (2.19, 4.11)	2.30 (1.67, 3.59)	0.019
Serum HDL-C (mmol/L)	1.21 (0.99, 1.49)	1.20 (0.99, 1.57)	1.22 (0.93, 1.37)	0.417
Serum albumin (g/L)	37.00 (32.80, 41.10)	36.80 (32.10, 40.65)	37.40 (32.90, 41.30)	0.495
Serum phosphate (mmol/L)	1.23 (1.09, 1.42)	1.20 (1.05, 1.39)	1.34 (1.12, 1.47)	0.033
Serum calcium (mmol/L)	2.15 (2.05, 2.23)	2.18 (2.10, 2.27)	2.05 (2.01, 2.13)	<0.001
iPTH (pg/mL)	56.50 (50.09, 105.00)	51.00 (41.98, 68.43)	105.00 (65.81, 225.90)	<0.001
UTP (g/24h)	1.26 (0.51, 3.12)	1.32 (0.38, 3.36)	1.02 (0.58, 2.27)	0.569
CRP(mg/L)	3.00 (1.75, 5.00)	3.00 (1.75, 5.00)	3.00 (1.25, 4.75)	0.659
FFAs (mmol/L)	0.65 (0.49, 0.67)	0.62 (0.39, 0.65)	0.67 (0.65, 1.13)	<0.001
Hypertension history (yes/no)	84/19	55/13	29/6	0.807
Diabetes history (yes/no)	47/56	24/44	23/12	0.003
Hyperlipemia (yes/no)	69/34	44/24	25/10	0.492
Calcium-containing phosphorus binder use (yes/no)	23/80	13/55	10/25	0.275
Lipid-lowering drug use (yes/no)	57/46	35/33	22/13	0.271
Antiplatelet drug use (yes/no)	29/74	11/57	18/17	<0.001
Vitamin D use (yes/no)	16/87	9/59	7/28	0.369

eGFR, estimated glomerular filtration rate; AST, aspartate aminotransferase; ALT, alanine aminotransferase; HbA1c, hemoglobin A1c; LDL-C, low-density lipoprotein cholesterol; HDL-C, high-density lipoprotein cholesterol; iPTH, intact parathyroid hormone; UTP, 24 hours urine total protein; CRP, C reactive protein; FFAs, free fatty acids.

### Determination of independent associated factors for AAC severity

To identify associated factors for moderate-to-severe vascular calcification (AAC score≥5), we performed univariate binary logistic regression analysis on variables in **Table 1**. The variables that were significantly related to AAC severity in the univariate logistic regression analysis included age, SBP, hemoglobin, eGFR, serum creatinine, serum urea, serum total cholesterol, serum LDL-C, serum calcium, iPTH, FFAs, diabetes history and antiplatelet drug use. Variables with *P* < 0.1 and traditional and well-known risk factors in the univariate logistic regression analysis were entered into the multivariable logistic regression model. Among these factors, SBP, total cholesterol and serum LDL-C were not included due to the inclusion of hypertension and hyperlipemia histories. For kidney function, we selected eGFR as a representative marker because of the strong correlation and collinearity among eGFR, serum creatinine, and blood urea nitrogen. Therefore, the variables ultimately included in the multivariate regression analysis were age, hemoglobin, eGFR, ALT, fasting blood glucose, serum phosphate, serum calcium, iPTH, FFAs, history of hypertension, diabetes and hyperlipidemia as well as the use of calcium-containing phosphorus binder, lipid-lowering drugs, antiplatelet drugs, and vitamin D. Collinearity diagnostics showed that the tolerance of the independent variables in the model ranged from 0.446 to 0.888, and the variance inflation factor ranged from 1.126 to 2.244, indicating that there was no significant multicollinearity among the independent variables (**Table 2**). As **Table 3** shows, iPTH (OR = 1.019, *P* = 0.022, 95% CI:1.003-1.035) and FFAs (OR = 1.269, *P* = 0.012, 95% CI:1.053-1.528) were independent factors of AAC. Additionally, compared with patients without diabetes, those with diabetes exhibited a significantly higher associated factor for AAC (OR = 18.472, *P* = 0.014, 95% CI: 1.817–187.745). Likewise, compared with patients not using antiplatelet, those using antiplatelet also constituted an independently associated factor for AAC (OR = 9.613, *P* = 0.046, 95% CI: 1.036–89.187). However, the observed association between antiplatelet therapy and vascular calcification should be interpreted with caution, as antiplatelet use is more likely a marker of underlying cardiovascular comorbidity rather than a direct contributor to calcification progression. We also performed Spearman’s rank correlation analysis between eGFR and FFAs. The results showed a weak but statistically significant negative correlation (r = -0.214, *P* = 0.030), indicating that while eGFR and FFAs are related, the association is relatively modest. The Hosmer–Lemeshow test revealed no statistically significant difference between the observed probability of moderate-to-severe vascular calcification and the predicted probability derived from the multivariate regression model (χ² = 1.913, *P* = 0.984), with *P* > 0.05, indicating a good goodness-of-fit for the model.

**Table 2:** Collinearity diagnostics for the regression model.

Variables	Collinearity statistics	
	Tolerance	VIF
Age (years)	0.687	1.456
Hemoglobin (g/L)	0.446	2.244
eGFR(ml/min·1.73m^2^)	0.469	2.131
ALT (IU/L)	0.664	1.505
Fasting serum glucose (mmol/L)	0.723	1.383
Serum phosphate (mmol/L)	0.692	1.446
Serum calcium (mmol/L)	0.733	1.365
PTH (pg/mL)	0.601	1.664
FFA (mmol/L)	0.77	1.298
Hypertension history (yes/no)	0.784	1.276
Diabetes history (yes/no)	0.781	1.281
Hyperlipemia (yes/no)	0.769	1.301
Calcium-containing phosphorus binder use (yes/no)	0.888	1.126
Lipid-lowering drug use (yes/no)	0.644	1.552
Antiplatelet drug use (yes/no)	0.722	1.385
Vitamin D use (yes/no)	0.82	1.219

**Table 3:** Independent associated factors for AAC according to univariate and multivariate logistic regression analysis in patients with CKD stage 3-4.

Variables	Univariate			Multivariate		
	OR	95%CI	Sig	OR	95%CI	Sig
Age (years)	1.094	1.038-1.152	0.001	1.091	0.964-1.235	0.167
Male/female (n)	1.100	0.454-2.663	0.833	Not selected		
SBP(mmHg)	1.032	1.006-1.060	0.018	Not selected		
DBP(mmHg)	1.029	0.991-1.068	0.141	Not selected		
CO_2_CP(vol%)	0.972	0.921-1.026	0.306	Not selected		
Smoking history(yes/no)	1.608	0.707-3.657	0.257	Not selected		
Drinking history(yes/no)	1.125	0.438-2.888	0.807	Not selected		
Hemoglobin (g/L)	0.974	0.957-0.991	0.003	1.011	0.971-1.053	0.594
eGFR (ml/min·1.73m^2^)	0.954	0.922-0.986	0.005	1.014	0.932-1.104	0.748
Serum creatinine (μmol/L)	1.006	1.001-1.012	0.030	Not selected		
Serum urea (μmol/L)	1.104	1.035-1.177	0.003	Not selected		
AST (IU/L)	1.003	0.952-1.056	0.920	Not selected		
ALT (IU/L)	0.965	0.926-1.005	0.088	1.043	0.929-1.170	0.477
HbA1c (%)	0.906	0.691-1.189	0.478	Not selected		
Fasting serum glucose (mmol/L)	1.191	0.995-1.426	0.057	0.991	0.678-1.448	0.963
Serum uric acid (μmol/L)	1.002	0.998-1.006	0.289	Not selected		
Serum total cholesterol (mmol/L)	0.745	0.560-0.990	0.042	Not selected		
Serum triglycerides (mmol/L)	0.996	0.674-1.472	0.982	Not selected		
Serum LDL-C (mmol/L)	0.679	0.476-0.969	0.033	Not selected		
Serum HDL-C (mmol/L)	0.497	0.165-1.496	0.214	Not selected		
Serum albumin (g/L)	1.026	0.968-1.087	0.388	Not selected		
Serum phosphate (mmol/L)	2.399	0.833-6.911	0.105	3.454	0.158-75.456	0.431
Serum calcium (mmol/L)	0.016	0.001-0.279	0.005	0.100	0.001-8.553	0.311
iPTH (pg/mL)	1.013	1.006-1.020	<0.001	1.019	1.003-1.035	0.022
UTP (g/24h)	0.873	0.662-1.150	0.334	Not selected		
CRP (mg/L)	0.989	0.855-1.143	0.876	Not selected		
FFAs (mmol/L)	1.117	1.039-1.202	0.003	1.269	1.053-1.528	0.012
Hypertension history (yes/no)	1.142	0.393-3.320	0.807	0.194	0.014-2.738	0.225
Diabetes history (yes/no)	3.514	1.491-8.279	0.004	18.472	1.817-187.745	0.014
Hyperlipemia (yes/no)	1.364	0.562-3.308	0.493	6.286	0.697-56.736	0.101
Calcium-containing phosphorus binder use (yes/no)	1.692	0.654-4.377	0.278	5.715	0.489-66.777	0.165
Lipid-lowering drug use (yes/no)	1.596	0.693-3.675	0.272	0.736	0.087-6.197	0.778
Antiplatelet drug use (yes/no)	5.487	2.175-13.839	<0.001	9.613	1.036-89.187	0.046
Vitamin D use (yes/no)	1.639	0.554-4.852	0.372	4.932	0.304-80.06	0.262

eGFR, estimated glomerular filtration rate; AST, aspartate aminotransferase; ALT, alanine aminotransferase; HbA1c, hemoglobin A1c; LDL-C, low-density lipoprotein cholesterol; HDL-C, high-density lipoprotein cholesterol; iPTH, intact parathyroid hormone; UTP, 24 hours urine total protein; CRP, C reactive protein; FFAs, free fatty acids.

### Discriminatory ability of FFAs for AAC

**Figure 2:**
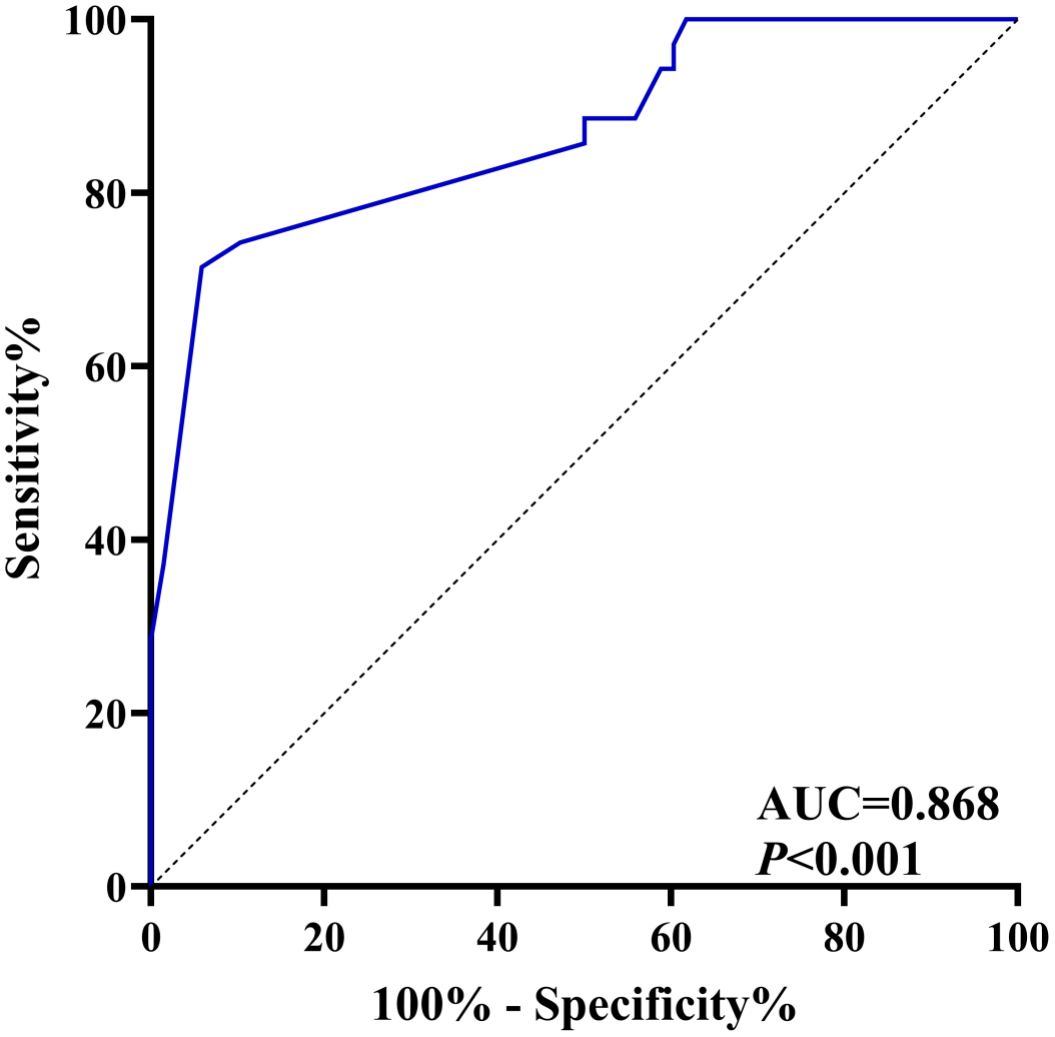
ROC curve analysis of serum FFA levels in AAC severity.

The discriminatory ability of serum FFAs for AAC was assessed by ROC curve analysis (**Figure 2**; **Table 4**). FFAs levels showed the biggest AUC of 0.868, with a sensitivity of 71.4% and a specificity of 94.1% and the optimal threshold was 0.665 mmol/L.

**Table 4:** ROC analysis of FFAs for discriminating AAC severity.

Variables	AUC (95%CI)	Cutoff	Sensitivity	Specificity
FFAs (mmol/L)	0.868(0.792,0.945)	0.665	71.4	94.1

FFAs, free fatty acids.

## Discussion

Our study has yielded the following three key findings: 1. In CKD patients, free fatty acids levels are positively correlated with vascular calcification. 2. Elevated levels of free fatty acids are an independent associated factor for moderate-to-severe vascular calcification. 3. When the serum free fatty acids level in CKD patients exceeds 0.665mmol/L, it serves as a discriminator for moderate-to-severe calcification.

As an important source of energy for the human body, free fatty acids are involved in various metabolic regulatory processes. Previous studies showed that FFAs were correlated with obesity (Sasaki et al., 2022; Liqiang et al., 2023), metabolic syndrome (Henderson, 2021), insulin resistance (Pereira et al., 2021), diabetes (Henderson, 2021; Li et al., 2024). Regarding cardiovascular diseases, Yuan *al* found that elevated FFA levels were independently associated with increased risks of all-cause death, cardiac death, and MACE (Yuan et al., 2023). Another research group (Xin et al., 2021) demonstrated that serum FFA levels were independently associated with severe coronary artery calcification (SCAC), and could have predictive capacity for SCAC. In the field of kidney diseases, there are relatively few clinical studies focusing on free fatty acids. Wu *al.* found that NEFA levels were independently associated with increased risk of cardiovascular events after correction for clinical confounding factors (Wu and Chen, 2021). Another research in a homogeneous population of older men with moderate CKD revealed that elevated serum FFAs was associated with cardiovascular disease mortality, and particularly with mortality due to acute myocardial infarction (Xiong et al., 2015). Our study firstly investigated the relationship between FFAs and vascular calcification in CKD patients.

A positive correlation was observed between circulating FFA concentrations and the severity of vascular calcification. Previous basic research findings may provide plausible explanations for this clinical phenomenon. A study on the potential role of short-chain fatty acids (SCFAs) in vascular calcification revealed that butyrate significantly enhanced high phosphate (Pi)-induced calcification and osteogenic phenotypic transformation of VSMCs *in vitro* experiments using established cellular and animal models of vascular calcification. Subsequent research further confirmed that butyrate markedly promotes calcification of *in vitro* cultured aortic rings under high-phosphate conditions, as well as vascular calcification induced by high-dose vitamin D3 (vD3) in mice. Mechanistic studies indicate that butyrate accelerates vascular calcification through a dual mechanism: inhibiting histone deacetylase (HDAC) activity and activating the NF-κB signaling pathway (Zhong et al., 2022). In addition, elevated FFAs may enhance fatty acid oxidation (FAO), which may also contribute to this process. Two studies investigating the role of mitochondria in VSMCs phenotypic switching demonstrated that upon transition to a synthetic phenotype, VSMCs exhibited increased fatty acid oxidation. Researchers hypothesized that this metabolic reprogramming may serve to provide additional energy to support VSMCs proliferation, migration, and extracellular matrix synthesis and secretion (Salabei and Hill, 2013; Scheede-Bergdahl and Bergdahl, 2017). Notably, although our Spearman correlation analysis revealed a statistically significant negative correlation between eGFR and FFAs, the modest strength of this association suggests that the relationship between renal function and FFAs levels is weak. Nevertheless, given their well-established physiological crosstalk in the context of CKD, the independent contribution of FFAs to the outcomes observed in our multivariate model should be interpreted with due consideration of their interrelationship with renal function. Future mechanistic studies are warranted to further disentangle the complex interplay between renal dysfunction and FFAs dysregulation.

In our study, the observed lower TC and LDL-C levels in the severe calcification group are intriguing. Although the proportion of lipid-lowering drug use did not differ significantly across groups, this does not exclude potential differences in treatment intensity, dosage, or adherence. Alternatively, these lipid alterations may partly reflect disease-related metabolic disturbances in advanced CKD, such as malnutrition and inflammation, which are known to be associated with lower cholesterol levels. Given the cross-sectional nature of our data, the underlying mechanisms cannot be definitively determined and warrant further prospective investigation. Furthermore, traditional risk factors for vascular calcification such as hypertension history failed to show statistically significant differences in our analysis, which might be due to the relatively small sample size of our study.

The present study has several limitations that should be acknowledged. First, the cross-sectional design precludes any causal inferences regarding the observed associations between FFAs and calcification. All laboratory parameters were measured concurrently, and therefore the temporal relationship between FFA levels and calcification cannot be determined. Second, the number of covariates included in our multivariable model—selected not only according to the results from univariate logistic regression analysis but also on the basis of clinically established factors for vascular calcification—resulted in a low events-per-variable ratio (approximately 2.2), which is below the commonly recommended threshold of 10 and may increase the risk of overfitting. Accordingly, our findings should be interpreted as exploratory, and the observed associations warrant validation in larger, independent cohorts before any definitive conclusions can be drawn. Third, the optimal cutoff value of 0.665 mmol/L was derived from our single-center cohort and should be considered exploratory. This threshold requires validation in larger, independent, and preferably multicenter prospective cohorts to confirm its generalizability and clinical utility. Additionally, serum FFA levels are influenced by multiple variables such as nutritional status, physical activity, and underlying diseases; thus, a single measurement may not fully capture their dynamic fluctuations. These constraints highlight the need for future large-scale studies to confirm our observations.

## Conclusion

Our results demonstrated a positive correlation between serum FFAs levels and vascular calcification severity in patients diagnosed with CKD stage 3-4. The FFAs levels were independently associated with the severity of vascular calcification in this population with a discriminatory ability of 0.665 mmol/L.

## Data Availability

The original contributions presented in the study are included in the article/supplementary material. Further inquiries can be directed to the corresponding author.
